# Preregistration nursing students’ perceived confidence in learning about patient safety in selected Kenyan universities

**DOI:** 10.4102/curationis.v42i1.1974

**Published:** 2019-07-18

**Authors:** Nickcy N. Mbuthia, Mary M. Moleki

**Affiliations:** 1 Department of Nursing Sciences, Pwani University, Kilifi, Kenya; 2 School of Social Sciences, University of South Africa, Pretoria, South Africa

**Keywords:** patient safety, nursing education, competencies, preregistration, confidence

## Abstract

**Background:**

Improvement of patient safety in Kenya depends on knowledgeable nurses who are equipped with the clinical safety and sociocultural competences of patient safety.

**Objectives:**

This study assessed the theoretical and practical learning of these competences as perceived by nursing students.

**Method:**

A cross-sectional descriptive study was conducted on 178 preregistration Bachelor of Nursing students from two Kenyan universities using the Health Professional Education in Patient Safety Survey. This tool assessed the students’ confidence in learning about clinical safety and the sociocultural aspects of patient safety in the classroom and clinical settings. Descriptive statistics summarised the sample and survey responses, while paired *t*-tests and ANOVA were used to compare responses across learning settings and year of study.

**Results:**

The students reported higher confidence about learning on the clinical aspects than on the sociocultural issues of patient safety with the lowest mean scores recorded in ‘Understanding human and environmental factors’ and ‘Recognising, responding and disclosing adverse events’. They reported significantly higher confidence scores in the classroom setting than the clinical setting with no significant difference in reported confidence across the years of study. They were less confident in speaking up about patient safety issues in the clinical areas with 52.2% feeling that reporting a patient safety problem will result in negative repercussions.

**Conclusion:**

Nursing programmes in Kenya need to reinforce the sociocultural aspects of patient safety in the curriculum. The patient safety culture in the clinical placements sites needs to be conducive to enable, and not hinder, the acquisition of these competences.

## Introduction

Patient safety has emerged as one of the most pressing challenges in health-care systems around the world, mainly because of the increasing complexity of the health delivery systems (Jha et al. 2013; Landrigan et al. 2010; Wilson et al. 2012). The foundation of patient safety is in the way the health-care professionals are trained and educated to be able to deliver safe and quality care (European Commission 2014; Institute of Medicine 2007). According to the World Health Organization (WHO), education has been underused and undervalued as a vital tool for addressing the challenges of achieving improved patient safety (WHO 2011). Admittedly, nurses follow an extensive education before they are allowed to diagnose, treat and care for patients, but, with the evolving nature of health-care, more needs to be done concerning patient safety.

The extent to which this education can prepare them for patient safety concepts is still questionable (Cresswell et al. 2013). According to Frenk et al. (2010), nursing education is not keeping up with the health-care changes and its complexities; hence, it produces ill-equipped graduates who are not able to deal with these new health-care challenges.

Patient safety education is competency-based, meaning that the student must be engaged and active in all aspects of acquiring the knowledge, skills and professional behaviours (Pijl-Zieber et al. 2014). The WHO in the ‘Patient Safety Curriculum Guide: Multi-professional Edition’ recommends that the health-care profession should be able to acquire the clinical safety competences as well as the core and broader sociocultural patient safety competences (WHO 2011). Clinical safety competences include medication safety, infection prevention and control, surgical safety and general patient safety. Sociocultural patient safety competences include but are not limited to patient-centred care, teamwork and collaboration, evidence-based practice, quality improvement, safety and informatics.

Nursing education in Kenya and the world over has mainly focused on the development of nurses who can provide patient care to the population. However, with the changing times, nursing education needs to shift its focus not only to care provision but also to the development of competences related to patient safety and quality improvement. Nursing educators are encouraged to integrate patient safety competences explicitly in their programmes and to ensure that the student gets the necessary theoretical and practical preparation related to these competences (Cronenwett et al. 2007; WHO 2011). Also, it is imperative that the perspectives of the student nurses, as well as graduate nurses, regarding how well the curriculum equips them with the necessary patient safety competences, are evaluated to ensure that the nursing education can prepare a competent and safe nurse (Dolansky & McMeekin 2015). Based on these evaluations, the educators can make significant revisions of the curriculum and assess the effect of the classroom and clinical environments in the acquisition of the competences (Lucian Leape Institute 2012). Many tools have been designed to measure the safety competences of health-care professionals; however, they do not necessarily measure all the aspects of patient safety competences (Okuyama et al. 2011). To measure the nurses and other health professionals’ self-reported patient safety competences, Ginsburg et al. (2012) developed the Health Professional Education in Patient Safety Survey (H-PEPSS), which is a tool that focuses on the sociocultural aspects of patient safety both in the classroom and clinical settings.

Many studies have been carried out to evaluate the nursing students’ self-reported patient safety competences in the upper-income countries (Colet et al. 2015; Doyle et al. 2015; Raymond, Medves & Godfrey 2017; Stevanin et al. 2015). However, studies in the low- and middle-income countries are limited, more so in the sub-Saharan region where no published study was identified during the literature search. The purpose of this study, therefore, is to add to the body of knowledge on patient safety education with perspectives about patient safety competences by nursing students from Kenya. Specifically, the objectives of the study were to (1) assess the nursing students’ perceived confidence in learning about patient safety in the classroom and clinical settings, (2) examine the differences in self-reported confidence in patient safety learning according to year of study and (3) assess the perceptions of nursing students about how broader patient safety issues are addressed in health professional education and comfort in speaking up about patient safety.

## Materials and methods

### Study design

A cross-sectional descriptive study using a quantitative approach was conducted to assess the perceptions of nursing students on patient safety knowledge and competences in two Kenyan universities.

### Study setting

The study was conducted in two public universities located in the Coastal and Western regions of Kenya.

### Population and sampling

The study population was preregistration Bachelor of Science in Nursing students in the second, third and fourth year levels of study in the two universities enrolled for the 2016–2017 academic year. The first year students were excluded from the study because they had not attended any clinical placement yet. A sample size of 194 was calculated from a population of 321 students with a 95% level of confidence, 0.05% margin of error and 10% nonresponse rate. A stratified random sampling technique was utilised to get a representative sample, by first grouping the population into strata according to the level of study.

### Study instrument

The H-PEPSS was used to collect data in this study after obtaining written permission from the developers of the tool. The H-PEPSS is a survey tool entrenched in a patient safety competency framework, focusing mainly on the sociocultural aspects of patient safety including culture, teamwork, communication, managing risk and understanding human factors. It was initially designed to measure health professionals’ self-reported patient safety competences around the time of entry to practice. However, its usage in other studies has shown that it is a valid tool for evaluating gaps in patient safety knowledge and competences in health professional students, thus helping the students reflect on patient safety-related issues (Ginsburg et al. 2012; Stevanin et al. 2015).

The instrument had two sections: Part A – Demographic Information: The respondent provided demographic information about themselves. Part B – The H-PEPSS. The H-PEPSS is further divided into three sections.

Section one contains 27 items that assess the students’ confidence in learning about the six sociocultural dimensions of patient safety and the clinical aspects of safety (safe clinical practice in general, hand hygiene, infection control and safe medication practices). According to the developers of the tool, the inclusion of the section on clinical aspects of safety serves the purpose of helping the respondents to differentiate between the clinical and sociocultural features of patient safety hence shifting their focus to the latter (Ginsburg et al. 2012). Each item begins with the stem ‘I feel confident in what I learnt about …’ and the respondents are asked to respond separately about their confidence in what they learnt in the classroom setting versus the clinical setting. Section two assesses the perceptions of students on how patient safety issues are addressed in the health professional education, while Section three measures how comfortable they are speaking up about patient safety. All items were answered using a 5-point disagree–agree Likert type scale and include a ‘neutral or unsure’ option.

The construct validity of the H-PEPSS was established by the developers of the tool using the confirmatory factor analysis (CFA). The developers explained that it was a better measure as it represented a measurement model which depicted the links between latent variables (the six patient safety competence dimensions) and their observed measures, that is, the 23 items used to measure these six dimensions. The internal consistency reliability of the six dimensions of patient safety competences was also established by the developers using Cronbach’s alpha coefficients, with it exceeding 0.80 for all six dimensions (Ginsburg et al. 2012).

### Data collection procedure

The study instrument, which was self-administered, was distributed to the participants during their break times from class. They were informed that the completion of the questionnaire was voluntary, and refusal to participate was not punishable. Those who were willing to participate were given an opportunity to read and understand the questionnaire information and instructions on the front page of the tool and then ask any questions. If they agreed, they signed and dated the consent. They were allowed privacy and time to complete the questionnaire.

### Data management and analysis

All statistical analysis was performed with IBM SPSS version 21. Demographic data were examined with frequency counts and percentages. Mean and standard deviation (SD) scores were calculated and analysed for each patient safety dimension by averaging the items in each dimension for each learning setting separately.

Paired *t*-test was used to examine the differences in self-reported confidence in patient safety learning in the classroom setting compared to learning in the clinical setting (two-tailed test). Also, a Cohen’s *d* effect size was calculated for each dimension to quantify the difference between the two settings. The researcher used Cohen’s suggestions on the interpretation of the effect size where *d* = 0.2 is considered a ‘small’ effect size, 0.5 represents a ‘medium’ effect size and 0.8 represents a ‘large’ effect size (Sullivan & Feinn 2012). One-way ANOVA test was used to examine the relationship of the year of study, and H-PEPSS mean scores and Tukey’s *post hoc* tests were conducted to identify the differences when the ANOVAs were significant. P-values were reported to two decimal places with values less than 0.01 being reported as 0.00. A *p*-value of less than 0.05 was considered statistically significant.

### Ethical considerations

Ethical clearance was obtained from the University of South Africa (HSHDC/386/2014) and Pwani University (ERC/PhD/008/2016). The researcher also got permission to conduct the study from the respective universities in which the study was being conducted. A participant informed consent form was attached with each survey tool that outlined the purpose of the research, the identity of the researcher, the role of the participant, the anticipated risks and benefits, and the assurance of anonymity, confidentiality and voluntary participation.

## Results

### Demographic characteristics

The response rate for the questionnaire was 91.8%, with 178 questionnaires completed and returned by participants. The demographic characteristics consisted of age, gender, university and year of study, which are summarised in Table 1. The mean age of the respondents was 22.1 years (± 1.45). Out of the 178 respondents, 91 (51.1%) were male, while 87 (48.9%) were female. The respondents from University A were 95 (53.4%) and from University B were 83 (46.6%). Also, 42 (23.6%) were in the second year, 83 (46.6%) were in the third year and 53 (29.8%) were in the fourth year of study.

**TABLE 1:** Demographic characteristics of the respondents (*n* = 178).

Variable	Frequency (*N*)	Percentage (%)
**Gender**		
Male	91	51.1
Female	87	48.9
**University**		
University A	95	53.4
University B	83	46.6
**Year of study**		
Second	42	23.6
Third	83	46.6
Fourth	53	29.8

Age: mean = 22.1; standard deviation = 1.45.

### Specific patient safety competence dimensions

The comparison of the mean scores on the self-reported competences in learning patient safety between the classroom and clinical settings among the nursing students was examined (Table 2).

**TABLE 2:** Self-reported patient safety competence in different learning settings.

Dimension	Learning setting	*N*	Mean	SD	*t*	*p*	Cohen’s *d*	Agreed or strongly agreed	
								Frequency (*N*)	Percentage (%)
Clinical safety	Classroom	174	4.00	0.79	1.93	0.06	0.10	140	80.5
	Clinical	177	3.92	0.79				138	78.3
Working in teams with other health professionals	Classroom	176	3.70	0.78	4.37	0.00*	0.28	112	64.0
	Clinical	174	3.48	0.85				94	54.2
Communicating effectively	Classroom	178	4.03	0.93	4.43	0.00*	0.31	136	76.6
	Clinical	178	3.75	0.96				113	63.5
Managing safety risks	Classroom	178	3.75	0.90	3.00	0.00*	0.21	120	67.2
	Clinical	177	3.56	0.94				105	59.5
Understanding human and environmental factors	Classroom	178	3.69	0.85	3.62	0.00*	0.28	112	63.2
	Clinical	178	3.44	0.91				94	52.7
Recognising, responding to and disclosing adverse events	Classroom	177	3.69	0.80	4.83	0.00*	0.30	114	64.4
	Clinical	177	3.45	0.85				95	53.5
Culture of safety	Classroom	176	3.76	0.79	4.30	0.00*	0.25	119	67.5
	Clinical	177	3.55	0.90				104	58.9

SD, standard deviation.

* , *p* < 0.001

In the classroom setting, the students reported the highest confidence in what they learnt in *Communicating effectively* and *Clinical safety* dimensions with mean scores of 4.03 (± 0.93) and 4.00 (± 0.79), respectively. They were least confident about what they learnt in *Understanding human and environmental factors* and *Recognising, responding and disclosing adverse events* with mean scores of 3.69 (± 0.8) on both dimensions. In the clinical setting, they demonstrated the same pattern as the classroom setting. The students reported highest confidence in what they learnt in *Clinical safety* and *Communicating effectively* dimensions with mean scores of 3.92 (± 0.79) and 3.75 (± 0.96), respectively. They reported the least confidence in *Recognising, responding and disclosing adverse events* and *Understanding human and environmental factors* with mean scores of 3.45 (± 0.85) and 3.44 (± 0.91), respectively. The paired *t*-tests for the dimensions indicated that there was a statistically significant decrease in the mean scores of the dimensions from the classroom setting to the clinical setting in all the dimensions except the *Clinical safety* dimension (*p* = 0.06). The Cohen’s *d* for those dimensions with a significant difference indicated that there was a small to moderate size effect ranging from 0.21 to 0.31, thus showing that learning in the classroom setting increased confidence in these dimensions to a greater extent as compared to the clinical setting.

The relationship between the year of study and their self-reported patient safety competence was also assessed. The patient safety dimension mean scores for the classroom and clinical settings by the year of study are reported in Table 3, which showed that in all the years, the mean scores for all the dimensions were higher in the classroom setting than the clinical setting. There were no statistical significances (*p* > 0.05) in the reported mean scores in the seven dimensions across the 3 years for the classroom and the clinical settings.

**TABLE 3:** Relationship between the year of study and self-reported patient safety competence.

Dimension	Setting	Year of study						*F*	*p*
		Second		Third		Fourth			
		Mean	SD	Mean	SD	Mean	SD		
Clinical safety	Class	3.96	0.77	3.93	0.80	4.14	0.79	1.18	0.31
	Clinical	3.85	0.72	3.85	0.78	4.15	0.83	2.68	0.07
Working in teams with others	Class	3.70	0.67	3.73	0.84	3.67	0.79	0.08	0.93
	Clinical	3.53	0.81	3.47	0.84	3.48	0.90	0.08	0.93
Communicating effectively	Class	3.94	0.72	4.10	0.85	4.00	1.17	0.44	0.65
	Clinical	3.75	0.81	3.70	1.04	3.81	0.96	0.22	0.80
Managing safety risks	Class	3.82	0.78	3.73	0.92	3.75	0.98	0.14	0.87
	Clinical	3.51	0.98	3.55	0.90	3.62	1.00	0.19	0.83
Understanding human and environmental factors	Class	3.56	0.77	3.68	0.84	3.82	0.93	1.12	0.33
	Clinical	3.27	0.92	3.44	0.89	3.59	0.92	1.47	0.23
Recognise, respond to and disclose adverse events	Class	3.57	0.74	3.75	0.81	3.70	0.84	0.77	0.47
	Clinical	3.26	0.83	3.45	0.88	3.59	0.79	1.77	0.17
Culture of safety	Class	3.67	0.71	3.82	0.83	3.75	0.77	0.54	0.59
	Clinical	3.55	0.78	3.60	0.90	3.52	1.00	0.11	0.89

SD, standard deviation.

### Broader patient safety issues and comfort speaking up about patient safety

The perceptions of students on how the broader patient safety issues are addressed in the health professional education as well as their comfort in speaking up about patient safety were assessed. The overall mean score for each item and the frequency counts for those who agreed or strongly agreed are summarised in Table 4.

**TABLE 4:** Broader aspects of patient safety and comfort in speaking up about patient safety.

Item	Mean	SD	Agree or strongly agree	
			Frequency (*n*)	Percentage (%)
**How broader patient safety issues are addressed in health professional education**				
As a student, the scope of what was ‘safe’ for me to do in the practice setting was very clear to me	3.67	0.93	117	66.1
There is consistency in how patient safety issues were dealt with by different preceptors in the clinical setting	3.25	1.09	82	46.3
I have sufficient opportunity to learn and interact with members of interdisciplinary teams	3.38	1.03	89	51.7
I have gained a solid understanding that reporting adverse events and close calls can lead to change	3.74	1.02	118	67.0
Patient safety is well integrated into the overall programme	3.53	0.99	99	56.9
Clinical aspects of patient safety are well covered in the programme	3.85	0.94	122	68.9
‘System’ aspects of patient safety are well covered in our programme	3.67	0.92	115	65.0
**Comfort speaking up about patient safety**				
In clinical settings, discussion around adverse events focuses mainly on system-related issues	3.42	1.00	93	52.5
In clinical settings, reporting a patient safety problem will result in negative repercussions for me	3.33	1.07	92	52.3
If I see someone engaging in unsafe care practice in the clinical setting, I feel safe to approach them	3.46	1.13	100	56.5

SD, standard deviation.

Regarding the broader patient safety issues, most of the students (69%, *n* = 122) were in agreement that the clinical aspects of patient safety are well covered by the nursing programme, with a mean score of 3.85 (SD ± 0.94). Conversely, less than half (46.3%, *n* = 82) of the students were in agreement that there is consistency in how patient safety issues were dealt with by different preceptors in the clinical setting. The confidence mean score was also low for the item at 3.25 (SD ± 1.09).

Regarding comfort on speaking up about patient safety in the clinical settings, 93 students (52.5%) agreed that the discussion around adverse events focused mainly on system-related issues instead of the individual most responsible for the event with a mean confidence score of 3.42 (± 1.00). Ninety-two students agreed (52.2%) that reporting a patient safety problem will result in negative repercussions for them (mean score 3.33 ± 1.07), while 100 (56.4%) of the students reported that they felt safe approaching someone whom they observed engaging in unsafe care practice (mean score 3.46 ± 1.13).

## Discussion

From the extensive literature review, this is the first study of this nature carried out to assess university nursing students’ perceived confidence in learning about patient safety in the classroom and the clinical settings. The students in this study reported high confidence in learning about *Clinical safety* and *Communicating effectively* in both settings which were consistent with results reported in similar studies.

Raymond et al. (2017) and Usher et al. (2017) in their studies indicated that the dimensions that the students were most confident about in their learning were *Clinical safety* and *Communicating effectively,* both in the clinical and classroom settings. These findings were supported by an earlier study by Duhn et al. (2012) who also reported higher student confidence in *Clinical safety*. Duhn et al. further went on to postulate that this was the case because the preregistration nursing curriculum usually reinforces the clinical aspects of patient safety (hand hygiene, infection control and medication safety) more than the sociocultural and organisational aspects of patient safety which include culture, teamwork, communication, managing risk and understanding human factors.

The results also show that students were more confident in learning about patient safety in the classroom setting than in the clinical setting, which validated the theory–practice gap in nursing education. Furthermore, this reported confidence by the students on learning patient safety in the classroom settings than in the clinical ones, confirms the results of other studies which reported the same finding (Colet et al. 2015; Doyle et al. 2015; Stevanin et al. 2015). As noted in the literature, the clinical setting is multifarious with many underlying factors that may influence learning as compared to the classroom setting, thus making learning more difficult for the students (Forber et al. 2015). Also, the students may not get enough clinical teaching from the preceptors who have other competing roles and are therefore not able to dedicate quality time to teaching the students (Madhavanpraphakaran, Shukri & Balachandran 2014). The theory–practice gap in nursing education has been reported in various studies, where it has been published that one of the challenges in nursing education is bridging the discrepancy between what is taught in the classroom and the actual realities in the clinical practice sites (Dadgaran, Parvizy & Peyrovi 2012; Grealish & Smale 2011; Maginnis & Croxon 2010). To adequately ensure that the nursing students can translate the patient safety theoretical knowledge into the clinical setting and hence enhance learning, faculty need to recognise that the gap exists and they need to implement teaching strategies which engage with practical realities in preparing safety-conscious nurses. One of the strategies suggested by Pauly-O’Neill and Cooper (2013) is the use of a clinical skills laboratory where the students are provided with a controlled environment through simulation, where they can develop and practise skills that they would otherwise have not had an opportunity to practise in real-life clinical settings. During the simulations, patient safety competences are infused with each experience, and this allows for the development of multiple competences, including the patient safety competences.

Regarding the students’ perceptions of broader patient safety issues, the item that the students were most confident about was on the coverage of the clinical aspects of patient safety (hand hygiene, transferring patients, medication safety) by the nursing programme. This finding was consistent with the results of the high mean score reported on the *Clinical safety* dimension. The item that the students indicated they were least confident about was on how patient safety issues were dealt with by the different preceptors in the clinical areas, a finding that was consistent with the findings by Lukewich et al. (2015) in a Canadian study. The perception of inconsistencies by the preceptors in clinical teaching could explain the result that the students felt more confident in what they learnt in the classroom than in the clinical settings. The role of the preceptor and the challenges that influence the position that could lead to inconsistencies in the way they deliver teaching to the students are well documented in the literature (Chang et al. 2015; Madhavanpraphakaran et al. 2014). Some of the characteristics of the preceptor that have been reported in relation to teaching patient safety are the patient safety practices of the preceptors themselves as well as the previous experience of the preceptor (Mansour 2013).

In the section on students’ perceptions on comfort in speaking up about patient safety in the clinical settings, the results of the first and second items in this section were indicative of the patient safety culture of the clinical settings which supported the findings in the dimension *Culture of safety.* In this dimension, the students reported a mean score of 3.55 in learning about this dimension within the clinical setting. This finding implies that the culture of the clinical setting, which forms part of the hidden curriculum that the students are exposed to, influences the students’ learning about patient safety, hence the low mean score on items related to patient safety culture. A patient safety culture is one which instead of blaming individuals for errors uses the errors as opportunities to improve the system and prevent harm (Ulrich & Kear 2014). A key element in establishing a safety culture is the reporting and speaking up about any concerns on patient safety by the staff as well as the students. As reported in this study, over half of the students agreed that reporting a patient safety problem would result in negative repercussions for them.

Further, only 56.4% of the students agreed that they felt safe approaching someone who they observed engaging in unsafe care practice. The findings of this study are consistent with the literature that shows that underreporting and failure of raising concern about errors is a prevalent issue in many organisations, both by the students as well as by the staff (Andrew & Mansour 2014). The Council of Deans of Health conducted a literature review related to supporting nursing, midwifery and allied health professional students in the United Kingdom to raise concerns about patient safety. Three key themes that emerged from the evidence were: students need to be empowered and supported so as to voice concerns about safety issues; there are specific patient safety issues that students consider as more dangerous thus prompting them to speak up; and the student may fail to report an incident fearing that it would impact the clinical assessment outcomes (Council of Deans of Health 2016).

This study had some strengths and limitations, which were acknowledged and taken into account. The strength of this study was the use of an instrument that has been validated and used in numerous studies on various health professionals both for preregistration students and for post-registration graduates. However, notably, there has been a limited number of studies carried out in low- and middle-income countries using the H-PEPSS or of this nature. The limitations of the study are related to the small number of universities used for the survey considering that there are more than 20 accredited degree nursing programmes in Kenya, therefore limiting the generalisability of the findings. However, the high response rate strengthens the results of this study.

## Conclusions

Patient safety education is a vital ingredient in ensuring the safety of our health systems. Nurses are at the forefront of care delivery, and therefore it is essential that they are equipped with the necessary competences to ensure that they can do so safely and competently. This study shows that the degree nursing students were more confident in learning about patient safety in the classroom setting than in the clinical setting. This indicated that there was an issue with the application of the theoretical aspects in the practical context, illuminating the theory–practice gap in nursing education. Consequently, the complexity of the clinical setting and the underlying factors that can hinder student learning cannot be ignored. Nursing faculty needs to establish stronger and supportive links with the clinical sites to ensure both are clear on the expected outcomes of the curriculum and therefore bridge the gap between the classroom and clinical learning.

Students were also more confident about the clinical aspects of patient safety and less confident about the sociocultural elements of patient safety, which are core competences that a nurse should have to be patient safety competent. Moreover, the students reported that the patient safety aspects were not sufficiently covered in their programmes and they feared speaking up about patient safety in the clinical areas. Faculty and clinical educators need to review the nursing programmes and integrate the sociocultural aspects of patient safety in the curriculum in both the classroom and the clinical settings. By teaching the students on these aspects, the students understand the proper patient safety culture and therefore will be aware that errors rarely occur in isolation but instead are multifaceted, and this, thus, gives them the courage to speak up about safety issues.

## References

[CIT0001] Andrew S. & Mansour M, 2014, ‘Safeguarding in medication administration: Understanding pre-registration nursing students’ survey response to patient safety and peer reporting issues’, *Journal of Nursing Management* 22(3), 311–321. 10.1111/jonm.12134 23919661

[CIT0002] Chang C.C., Lin L.M., Chen I.H., Kang C.M. & Chang W.Y, 2015, ‘Perceptions and experiences of nurse preceptors regarding their training courses: A mixed method study’, *Nurse Education Today* 35(1), 220–226. 10.1016/j.nedt.2014.08.002 25175623

[CIT0003] Colet P.C., Cruz J.P., Cruz C.P., Al-Otaibi J., Qubeilat H. & Alquwez N, 2015, ‘Patient safety competence of nursing students in Saudi Arabia: A self-reported survey’, *International Journal of Health Sciences* 9(4), 418–426. 10.12816/0031231 26715921 PMC4682596

[CIT0004] Council of Deans of Health, 2016, *Supporting nursing, midwifery and allied health professional students to raise concerns with the quality of care*, Council of Deans of Health, London. 10.1016/j.nedt.2017.06.006 28711721

[CIT0005] Cresswell K., Howe A., Steven A., Smith P., Ashcroft D., Fairhurst K. et al., 2013, ‘Patient safety in healthcare preregistration educational curricula: Multiple case study-based investigations of eight medicine, nursing, pharmacy and physiotherapy university courses’, *BMJ Quality & Safety* 22(10), 843–854. 10.1136/bmjqs-2013-001905 23728120

[CIT0006] Cronenwett L., Sherwood G., Barnsteiner J., Disch J., Johnson J., Mitchell P. et al., 2007, ‘Quality and safety education for nurses’, *Nursing Outlook* 55(3), 122–131. 10.1016/j.outlook.2007.02.006 17524799

[CIT0007] Dadgaran I., Parvizy S. & Peyrovi H, 2012, ‘A global issue in nursing students’ clinical learning: The theory-practice gap’, *Procedia – Social and Behavioral Sciences* 47(1), 1713–1718. 10.1016/j.sbspro.2012.06.888

[CIT0008] Dolansky M.A. & McMeekin D, 2015, ‘Quality and safety: QSEN’, in Smith M., Carpenter R. & Fitzpatrick J. (eds.), *Encyclopedia of nursing education*, pp. 283–285, Springer Publishing, New York.

[CIT0009] Doyle P., VanDenKerkhof E.G., Edge D.S., Ginsburg L. & Goldstein D.H, 2015, ‘Self-reported patient safety competence among Canadian medical students and postgraduate trainees: A cross-sectional survey’, *BMJ Quality & Safety* 24(2), 135–141. 10.1136/bmjqs-2014-003142 PMC4316835 25605953

[CIT0010] Duhn L., Karp S., Oni O., Edge D., Ginsburg L. & VanDenKerkhof E, 2012, ‘Perspectives on patient safety among undergraduate nursing students’, *Journal of Nursing Education* 51(9), 526–531. 10.3928/01484834-20120706-04 22766076

[CIT0011] European Commission, 2014, *Key findings and recommendations on education and training in patient safety across Europe by the Patient Safety and Quality of Care Working Group*, European Commission, Brussels.

[CIT0012] Forber J., DiGiacomo M., Davidson P., Carter B. & Jackson D, 2015, ‘The context, influences and challenges for undergraduate nurse clinical education: Continuing the dialogue’, *Nurse Education Today* 35(11), 1114–1118. 10.1016/j.nedt.2015.07.006 26264968

[CIT0013] Frenk J., Chen L., Bhutta Z.A., Cohen J., Crisp N., Evans T. et al., 2010, ‘Health professionals for a new century: Transforming education to strengthen health systems in an interdependent world’, *The Lancet* 376(9756), 1923–1958. 10.1016/S0140-6736(10)61854-5 21112623

[CIT0014] Ginsburg L., Castel E., Tregunno D. & Norton P.G, 2012, ‘The H-PEPSS: An instrument to measure health professionals’ perceptions of patient safety competence at entry into practice’, *BMJ Quality & Safety* 21(8), 676–684. 10.1136/bmjqs-2011-000601 PMC3402748 22562876

[CIT0015] Grealish L. & Smale L.A, 2011, ‘Theory before practice: Implicit assumptions about clinical nursing education in Australia as revealed through a shared critical reflection’, *Contemporary Nurse* 39(1), 51–64. 10.5172/conu.2011.39.1.51 21955266

[CIT0016] Institute of Medicine, 2007, *Preventing medication errors*, National Academies Press, Washington, DC 10.17226/11623

[CIT0017] Jha A.K., Larizgoitia I., Audera-Lopez C., Prasopa-Plaizier N., Waters H. & Bates D.W, 2013, ‘The global burden of unsafe medical care: Analytic modelling of observational studies’, *BMJ Quality and Safety* 22(10), 809–815. 10.1136/bmjqs-2012-001748 24048616

[CIT0018] Landrigan C.P., Parry G.J., Bones C.B., Hackbarth A.D., Goldmann D.A. & Sharek P.J, 2010, ‘Temporal trends in rates of patient harm resulting from medical care’, *The New England Journal of Medicine* 363(22), 2124–2134. 10.1056/NEJMsa1004404 21105794

[CIT0019] Lucian Leape Institute, 2012, *Unmet needs: Teaching physicians to provide safe patient care*, Lucian Leape Institute, Boston, MA.

[CIT0020] Lukewich J., Edge D.S., Tranmer J., Raymond J., Miron J., Ginsburg L. et al., 2015, ‘Undergraduate baccalaureate nursing students’ self-reported confidence in learning about patient safety in the classroom and clinical settings: An annual cross-sectional study (2010–2013)’, *International Journal of Nursing Studies* 52(5), 930–938. 10.1016/j.ijnurstu.2015.01.010 25698119

[CIT0021] Madhavanpraphakaran G.K., Shukri R.K. & Balachandran S, 2014, ‘Preceptors’ perceptions of clinical nursing education’, *Journal of Continuing Education in Nursing* 45(1), 28–34. 10.3928/00220124-20131223-04 24369755

[CIT0022] Maginnis C. & Croxon L, 2010, ‘Transfer of learning to the nursing clinical practice setting’, *Rural and Remote Health* 10(2), 1313. 20462300

[CIT0023] Mansour M, 2013, ‘Examining patient safety education in pre-registration nursing curriculum: Qualitative study’, *Journal of Nursing Education and Practice* 3(12), 157–167. 10.5430/jnep.v3n12p157

[CIT0024] Okuyama A., Martowirono K. & Bijnen B, 2011, ‘Assessing the patient safety competencies of healthcare professionals: A systematic review’, *BMJ Quality & Safety* 20(11), 991–1000. 10.1136/bmjqs-2011-000148 PMC3203526 21880646

[CIT0025] Pauly-O’Neill S. & Cooper E, 2013, ‘Addressing gaps in quality and safety education during pre-licensure clinical rotations’, *Journal of Nursing Education and Practice* 3(11), 65–70. 10.5430/jnep.v3n11p65

[CIT0026] Pijl-Zieber E.M., Barton S., Konkin J., Awosoga O. & Caine V, 2014, ‘Competence and competency-based nursing education: Finding our way through the issues’, *Nurse Education Today* 34(5), 676–678. 10.1016/j.nedt.2013.09.007 24090616

[CIT0027] Raymond J.M., Medves J.M. & Godfrey C.M, 2017, ‘Baccalaureate nursing students’ confidence on patient safety’, *Journal of Nursing Education and Practice* 7(6), 56–64. 10.5430/jnep.v7n6p56

[CIT0028] Stevanin S., Bressan V., Bulfone G., Zanini A., Dante A. & Palese A, 2015, ‘Knowledge and competence with patient safety as perceived by nursing students: The findings of a cross-sectional study’, *Nurse Education Today* 35(8), 926–934. 10.1016/j.nedt.2015.04.002 25959704

[CIT0029] Sullivan G.M. & Feinn R, 2012, ‘Using effect size – Or why the P value is not enough’, *Journal of Graduate Medical Education* 4(3), 279–282. 10.4300/JGME-D-12-00156.1 23997866 PMC3444174

[CIT0030] Ulrich B. & Kear T, 2014, ‘Patient Safety and patient safety culture: Foundations of excellent health care delivery’, *Nephrology Nursing Journal* 41(5), 447–457. 26295088

[CIT0031] Usher K., Woods C., Parmenter G., Hutchinson M., Mannix J., Power T. et al., 2017, ‘Self-reported confidence in patient safety knowledge among Australian undergraduate nursing students: A multi-site cross-sectional survey study’, *International Journal of Nursing Studies* 71, 89–96. 10.1016/j.ijnurstu.2017.03.006 28364581

[CIT0032] Wilson R.M., Michel P., Olsen S., Gibberd R.W., Vincent C., El-Assady R. et al., 2012, ‘Patient safety in developing countries: Retrospective estimation of scale and nature of harm to patients in hospital’, *BMJ* 344(e832), 1–14. 10.1136/bmj.e832 22416061

[CIT0033] World Health Organization (WHO), 2011, *Patient safety curriculum guide: Multi-professional edition*, WHO, Geneva.

